# An Elderly Man with an Unusual Cause of Abdominal Pain and Urinary Retention

**DOI:** 10.34067/KID.0000000978

**Published:** 2026-03-26

**Authors:** Aditi Singh, C. Elena Cervantes, Mohamad Hanouneh

**Affiliations:** 1 Division of Nephrology, Department of Medicine, Johns Hopkins University School of Medicine, Baltimore, Maryland; 2 Nephrology Center of Maryland, Baltimore, Maryland

**Keywords:** AKI, kidney anatomy, bladder and urinary tract

## Abstract

**Figure:**
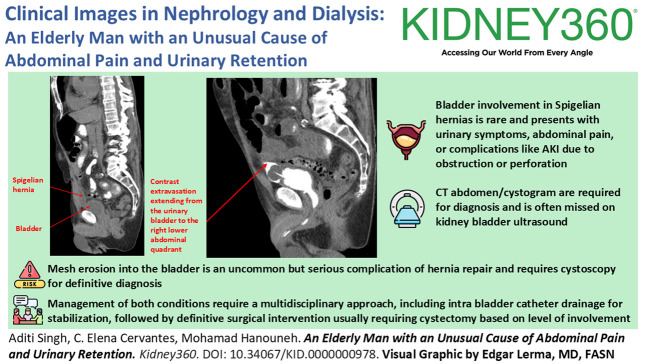

## Case Description

**Figure 1:**
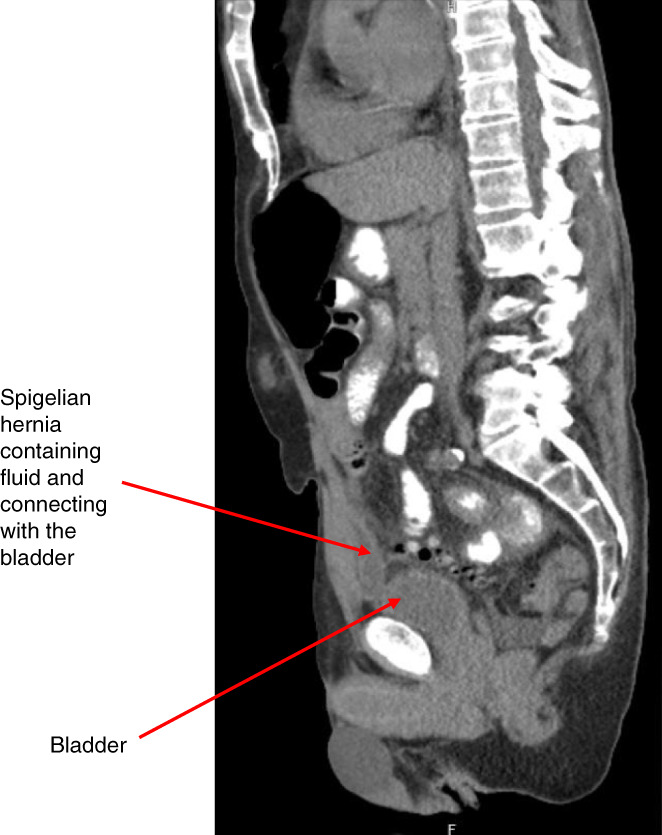
**CT of the abdomen without contrast.** CT, computerized tomography.

**Figure 2:**
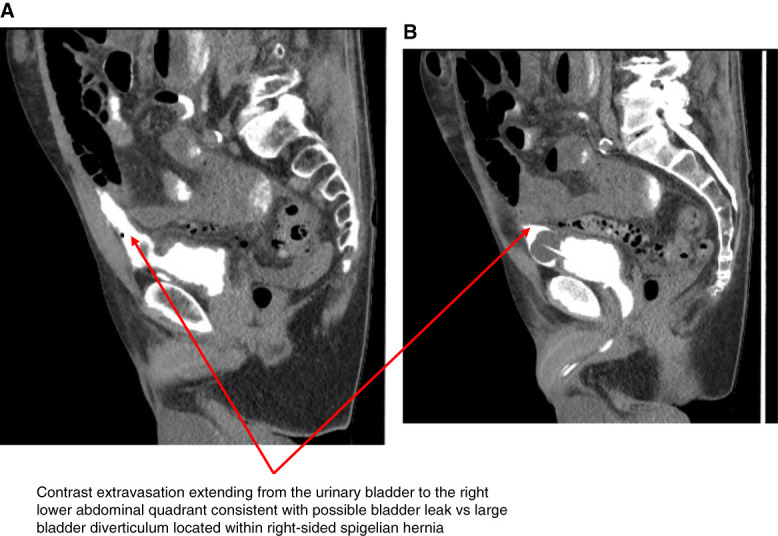
**CT cystography.** (A) Contrast extravasation extending from the urinary bladder to the right lower abdominal quadrant consistent with possible bladder leak versus (B) large bladder diverticulum located within right-sided spigelian hernia.

A 72-year-old man with a history of bladder carcinoma *in situ* treated with intravesicular BCG, bladder diverticulum, right inguinal hernia mesh repair, and recurrent urinary tract infections presented with abdominal pain and inability to urinate for 24 hours. His initial vital signs revealed a BP of 90/60 mm Hg, heart rate of 110 beats per minute, respiratory rate of 22 breaths per minute, and temperature of 37.3°C. Physical examination demonstrated suprapubic tenderness. Initial laboratory data revealed an AKI with a serum creatinine of 4.42 mg/dl (baseline 1.34 mg/dl) BUN of 40 mg/dl and white blood count 22.39 k/mm^3^. Urinalysis showed positive leukocyte esterase, >100 white blood count per high power field, and microscopic hematuria with 50–100 red blood cell per high power field. He was empirically initiated on piperacillin and tazobactam for possible urinary tract infection. Subsequent urine culture grew *Escherichia coli* and *Proteus mirabilis*. Kidney bladder ultrasound at presentation revealed mild bilateral hydronephrosis but grossly normal bladder with no evidence of retention. Kidney function continued to decline with a peak serum creatinine of 5.94 mg/dl. Computerized tomography (CT) without contrast of the abdomen identified a moderate right-sided Spigelian hernia containing fluid connected to the bladder (Figure 1). An indwelling catheter was placed with initial urinary output of 3 L/24 hours and improvement in renal function. Intraperitoneal fluid was collected with a fluid creatinine of 1.5 mg/dl not consistent with a urinary leak. A CT cystogram demonstrated contrast extravasation from the bladder into the hernia sac, raising concern for leak or diverticulum within the hernia (Figure 2, A and B). Cystoscopy confirmed a bladder diverticulum within the Spigelian hernia with intravesical mesh erosion. On discharge, the patient was managed with chronic catheterization and planned cystectomy with normalization of renal function.

## Discussion

Bladder involvement within Spigelian hernias is a rare finding, involving herniation of the bladder wall through the Spigelian aponeurosis, often presenting with abdominal pain or urinary tract symptoms such as frequency pain or urinary retention.^1^ Mesh erosion into the bladder after inguinal hernia repair is also an uncommon but serious complication.^2^ This case describes a rare complication of inguinal hernia repair leading to mesh erosion of a bladder diverticulum within a Spigelian hernia, leading to AKI that has thus far not been reported in the literature. Diagnosis is achieved through CT cystogram or cystoscopy and missed on routine kidney bladder ultrasounds. The large bladder diverticulum herniating into the Spigelian defect may have contributed to incomplete emptying without significant bladder distension, making retention less apparent on the initial kidney bladder ultrasound. The mild bilateral hydronephrosis noted was likely due to functional obstruction. The herniated bladder diverticulum acted as a low-resistance path, diverting urine away from the bladder lumen, causing ineffective bladder emptying, reduced functional bladder capacity, and elevated intravesical pressure. This pressure transmitted to the ureters led to the bilateral hydronephrosis noted and was relieved by indwelling catheterization. Definitive management requires surgical intervention, including hernia and diverticulum repair, removal of the eroded mesh, potentially necessitating cystectomy depending on the extent of bladder involvement.

## Teaching Points

- Bladder involvement in Spigelian hernias is rare and presents with urinary symptoms, abdominal pain, or complications such as AKI due to obstruction or perforation. CT abdomen/cystogram are required for diagnosis and are often missed on kidney bladder ultrasound.
- Mesh erosion into the bladder is an uncommon but serious complication of hernia repair and requires cystoscopy for definitive diagnosis.
- Management of both conditions requires a multidisciplinary approach, including intrabladder catheter drainage for stabilization, followed by definitive surgical intervention usually requiring cystectomy based on level of involvement.
